# Actionable recommendations from trainees to improve science training

**DOI:** 10.7554/eLife.59806

**Published:** 2020-08-07

**Authors:** Stephanie M Davis, Harinder Singh, Cara M Weismann, Adriana Bankston, Fátima Sancheznieto

**Affiliations:** 1 Future of ResearchPittsfieldUnited States; 2 Graduate Professional Success in STEM, University of California, IrvineIrvineUnited States; 3 School of Medicine and Public Health, University of Wisconsin MadisonMadisonUnited States

**Keywords:** research culture, mentorship, early-career researchers, science training, None

## Abstract

Over the past 20 years, a series of reports written by groups of senior researchers and administrators have recommended changes to improve the training environments for graduate students and postdoctoral researchers in the United States. However, academic institutions and departments have largely failed to implement these recommendations, which has exacerbated the problems faced by these trainees. Here, based on input from trainees at different career stages, we outline seven practical changes that academic institutions and departments can make to improve their training environments.

## Introduction

Building a productive and equitable scientific workforce requires widespread investment in training. Preparing graduate students and postdoctoral researchers (here collectively referred to as trainees) for successful careers requires training environments that foster inclusive and effective training practices. Currently, there is relatively little evaluation of the training provided by institutions in the United States, and there are few incentives to implement best practices. The National Academies of Science, Engineering, and Medicine (NASEM) and others have released multiple reports with recommendations on how to best rescue the academic training system from its flaws and crises (Alberts et al., 2014; NASEM, 2018a; NASEM, 2018b; NASEM, 2018c; NASEM, 2019). Unfortunately, a lack of buy-in by departmental and institutional leadership has led to no large-scale, concrete actions. Because of this, issues such as trainee burnout and systemic inequities remain unaddressed and widely spread (Ginther et al., 2011; Gibbs et al., 2016; Levecque et al., 2017; Nagy et al., 2019).

The recommendations in this article were originally developed at a meeting of trainees, faculty, academic administrators and leaders, and industry scientists, organized in June 2019 by Future of Research (a non-profit organization that champions, engages, and empowers early-career researchers). Inspired by the Declaration on Research Assessment (DORA), the TOP Guidelines developed by the Center for Open Science, and the Athena Swan Initiative, meeting attendees developed a set of nine training climate guidelines, each with bronze, silver, and gold tiers, that can be used by those wishing to implement change (https://mentoringfuturesci.net/). Here, we provide concrete actions based on six of these guidelines, plus one action regarding salary and benefits, and include examples of programs implementing them (Table 1). The other three guidelines from the meeting cover ‘Diversity and Inclusion Efforts', 'Transparent Accountability’ (for bullying and harassment), and ‘Mental Health and Wellness Resources’. Given the complexity and importance of each of these three areas, each merits independent discussion and is therefore not addressed in this article.

**Table 1.** Recommendations. Seven concrete actions for department leadership with examples.

Guideline	Examples	
1. Supplemental Mentorship	
Departments should require at least one other mentor figure beyond the main supervisor, or the creation of a mentorship committee for graduate students and postdocs.	The University of Michigan has recently piloted a mentorship committee program for postdocs (*M. Swanson, personal communication, May 2020).*
2. Peer Support	
Departments should facilitate peer cohorts for social support and peer mentorship, particularly where training start times are not synchronized, such as for postdocs.	The Department of Sociology at the University of Alabama at Birmingham provides all incoming graduate students with peer mentors. While UW-Madison and Brigham and Women's Hospital have postdoc peer mentorship opportunities, these are not incorporated into training/departmental programming.
3. Required Mentor Training	
Departments should require mentor training not merely as a compliance exercise, but as an investment into the professional development of their faculty, staff, and senior trainees.	All basic science graduate science programs at UCSF require faculty to participate "in at least one mentorship development activity of their choosing each year they have a student in their lab."
4. Exit Surveys	
Departments should require anonymous exit surveys from all trainees and staff, publishing aggregate data to ensure the transparent reporting of a department’s climate; diversity and inclusion efforts; bullying and harassment; and trainee mental health.	To date, 53 schools have signed onto the NGLS coalition to "collect and publish data using common standards on their life science training programs." We highlight the University of Northern Colorado for publishing thorough information on student satisfaction with the program, research advisor, and factors associated with choosing their field of study.
5. Clear Guidelines and Timelines	
Departments should provide graduate students and postdocs with clear guidelines and timelines, beyond grad student qualifying exams; the timing of career stage advancement should not solely depend on the main supervisor or thesis committee.	Universities in the United Kingdom, such as Oxford and UCL, have PhD thesis submission deadlines of 3–4 years. Albert Einstein College of Medicine has a committee that “reviews the progress of all students who have been in the program for five years or longer and requests an Exit Strategy from [them]".
6. Standard and Transparent Salary and Benefits	
Departments should provide trainees with benefits and salaries adjusted for the local cost of living, along with transparent and standardized benchmarks for raises based on years of training.	To our knowledge, the NIH Office of Intramural Training and Education is the only place in the United States that enforces standardized benefits and salary floors, adjusted for years of experience, for both PhDs and postdocs.
7. Career and Professional Development Resources	
Departments should require trainees to participate in career and professional development training and workshops of their choice, allowing for exploration of careers beyond academia.	While many schools provide career development opportunities, we highlight the Graduate School of Biomedical Science at UMass Medical School for their career development curriculum built into the PhD training program.

## Recommendations

### Supplemental mentorship

Trainees in the United States are normally expected to be reliant on a single person, who is likely providing guidance to other trainees, to be their sole mentor. While graduate students may sometimes receive informal mentorship from members of their dissertation committee, neither they nor postdocs are usually required by their programs or institutions to seek mentorship beyond their main supervisor. Requiring trainees to have a formal mentorship committee that encourages intentional consideration of their training needs serves multiple purposes. First, having multiple mentors provides trainees with several points of view and feedback on specific aspects of their training. The mentorship committee should contain mentors from other departments and from outside of academia, fostering external collaborations. Adding and removing mentors through deliberate conversation empowers trainees to articulate their specific mentorship needs as they evolve (Montgomery, 2017). Officially designating multiple mentors can also mitigate power imbalances in the relationship between the trainee and the primary supervisor should conflicts arise. For example, additional mentors may provide letters of recommendation if the supervisor threatens to withhold them. Finally, having multiple mentors reduces the mentorship load on the trainee’s primary supervisor, who is normally expected to meet all the trainee’s development needs (NASEM, 2019).

### Peer support

While faculty mentorship is a critical component of training, mentorship from peers may also cater to a trainee’s career, social and psychological needs. Unlike graduate students, postdoctoral researchers do not usually begin their positions at the same time. They therefore often lack the support of peer cohorts within their department, particularly if there are no postdoctoral associations on campus to provide a sense of community. Departments that group trainees into peer cohorts and provide trained and compensated staff to facilitate ongoing peer mentorship offer trainees the opportunity to reflect on their research progress and career development in a supportive environment. Working in groups creates a sense of community, expands trainees’ networks, and helps them cope with academic stress (Masefield, 2019). Departments can also provide funds for cohorts to socialize regularly and build community, provided the events are safe, inclusive, and accessible to everyone, including neurodiverse trainees and those with social anxiety.

### Required mentor training

Despite there being available data outlining mentorship best practices (Pfund et al., 2014), most departments continue to take a laissez-faire approach to the mentorship of future scientists. This leaves faculty to experiment with trainees as they implement the traditional ad-hoc apprenticeship model with little to no training. While mentor training has become more available in the past years, it is rarely compulsory: exceptions to this include Gilliam Fellowships awarded by the Howard Hughes Medical Institute and basic science departments at the University of California San Francisco (Table 1). Anecdotes from trainees, faculty, and staff at the June 2019 meeting suggest that many of the supervisors most in need of mentor training do not think they will receive any benefit from it, and therefore do not attend, despite studies showing training to be effective (Pfund et al., 2014). To incentivize buy-in, departments should either provide funds for faculty to attend training sessions or host on-site mentor training for all. Departments should recognize that requiring recurrent mentor training from their faculty is not merely an investment in the next generation of researchers, but also the career development of mentors, particularly those early in their careers. Trained mentors can list recurring mentor training in their CVs for grant applications, and on their group websites to recruit potential trainees.

### Exit surveys

Anonymous exit surveys can be used to effectively assess the climate of a training environment. Survey data can provide insights into the mental health and well-being of trainees, faculty, and staff; their perceptions of departmental commitment to diversity and inclusion; overall trainee satisfaction with a training program and supervisors; and prevalence of bullying and harassment. Public reports of survey data provide opportunities for leadership to send a clear message to the department about which problems most require addressing and which behaviors are not tolerated. Two caveats for surveys must be taken into account: first, studies have shown that on average, historically underrepresented faculty receive lower satisfaction ratings from students due to unconscious bias (MacNell et al., 2015; Fan et al., 2019). Second, care must be taken to avoid inadvertently ‘outing’ those reporting bullying, harassment, or other harmful behaviors, as the threat of retaliation against whistleblowers in academia is very real. The risk of survey data outing someone is higher in small departments and programs. Best practices for measuring incidences of harassment and abuse should be followed (NASEM, 2018a). De-identified survey data should be public and transparent to empower prospective trainees and hold administrative leadership accountable.

### Clear guidelines and timelines

Outside academic work settings, many organizations use tiered structured assessments and ratings to define expectations, provide performance feedback, and determine promotion potential. Within academia, there are similar skill development milestones required for degree conferral or completion of a fellowship. However, when a sole supervisor or committee is the arbiter in deciding when milestones are met, there is no accountability for ensuring trainees advance in their careers and are not retained due to internal bias, departmental politics, or exploitation for more publications. Ultimately, trainees deserve a transparent understanding of expectations. Specifically, trainees should be aware of what training they will be receiving beyond competency in their chosen field, as well as the duration of their training. Transparent communication of expectations can increase the efficacy of trainees, their productivity, and their commitment to a chosen career path. A clear set of guidelines ensures that trainees are trained consistently and that they have a realistic idea of the time required to acquire skills or achieve goals (see Table 1 for examples). Clear timelines for graduate students and postdocs, while field-specific, can provide a framework with which to hold supervisors and departments accountable for delivering appropriate training within a timeframe beneficial to the trainee’s career advancement.

### Standard and transparent salary and benefits

To prevent financial considerations from excluding trainees from pursuing academic careers, it is critical to provide them with clear information on salary and benefits available to them at prospective institutions. Given large gender pay disparities among graduate stipends and postdoctoral salaries across institutions in the United States (Athanasiadou et al., 2018), this information is particularly important for trainees from low-income backgrounds, for those in areas with a high cost of living, for those supporting families, and for those who are international scholars (McConnell et al., 2018). Inadequate salaries and benefits have led to a significant number of recent trainee-led protests to improve working conditions and compensation (Latimer and Horton, 2020). To this end, salaries and benefits should be adjusted for the local cost of living and include publicly available, standardized benchmarks from institutions related to minimum raises based on years of training. Departments should also have clear protections on trainee parental leave, sick leave, and vacation time, and provide affordable childcare for parents regardless of funding source or employee status. Finally, prospective trainees should also be informed of the potential loss of institutional benefits when obtaining certain fellowships or training awards (Ferguson et al., 2017). Available data on trainee compensation are scarce. This information should be publicly available, standardized by institutions, and clearly explained in both employment contracts and mentor/mentee compacts before the trainee joins a particular institution (NASEM, 2018c).

### Career and professional development resources

Over the years, the gap between the number of PhDs in science, technology, engineering, and mathematics (STEM) and available faculty positions has increased, with only 10–12% of STEM trainees transitioning to independent, tenure-track research careers in academia (NASEM, 2018b). This has resulted in a declining interest in academic careers among trainees and an increasing demand for better, more comprehensive career and professional development resources from their training programs (Fuhrmann, 2016). Additionally, while academic institutions benefit from the skills and talents of their trainees, these programs largely focus on preparing them for research-intensive faculty careers. Meanwhile, structured and intentional resources that prepare trainees for non-academic careers are often scarce. For trainees to smoothly transition into non-academic jobs, departments should support their trainees in exploring all available career choices early in their training through individual development plans (IDPs). Career exploration exercises should be followed by options for integrated internship opportunities and specific skill training, such as that provided by the School of Biomedical Sciences at UMass (Table 1). Because supervisors may not be supportive of trainees spending time ‘away from the bench’ (Fuhrmann, 2016), departments must incorporate these activities into their core training requirements. Departments should also track the career trajectories of their alumni and regularly and transparently report aggregate data on graduate career trajectories, as has been previously proposed by various groups and reports (Blank et al., 2017; NASEM, 2018c).

## Discussion

Several recently published reports point to a lack of transparency, sustainability, and equity in the STEM training landscape. One of them found that there is a high degree of overlap between recommendations from the last twenty-five years, and that the major obstacle to implementing these recommendations was a lack of buy-in and cooperation among departmental, institutional, state, and federal leadership (NASEM, 2018c). This reform gridlock and aversion to change has led to continued and widespread discontent among trainees. Trainee-led strikes and protests in the United States this past year (such as those at Harvard, UW-Madison, and the UC system; Latimer and Horton, 2020) and reports of dismal trainee mental health (Levecque et al., 2017; Nagy et al., 2019) are just two indicators of the current crisis. As recent and current trainees, we ask readers to think about the cost of graduate students and postdocs leaving the academic workforce en masse, particularly when most leaving are from underrepresented backgrounds. We also ask readers to consider the effect of those who remain in academic research spending cognitive and emotional energy fighting and reforming an inefficient system that prioritizes publications and grants over people.

Reluctance to commit to the reforms proposed here despite their alignment with recommendations that have worked at a smaller scale, has meant that the effectiveness of these changes remains untested. The feasibility of these recommendations is demonstrated by the programs and institutions that have already made a commitment to their trainees by implementing them (Table 1). Given the current pandemic and protests, the inequities of society at large have recently become more apparent to those who do not experience them, forcing programs and institutions to make changes previously thought unfeasible. Forward-thinking departments and institutions that revitalize their programs to more intentionally train the next generation of scientists will see almost immediate gains in their scientific output, and will also be better equipped to recruit and train from a more diverse pool of talented graduate students and postdocs in the future.

## Data Availability

All data generated or analyzed during this study are included in the manuscript and supporting files.
